# Non-Invasive Determination of Glucose Concentration Using a Near-Field Sensor

**DOI:** 10.3390/bios11030062

**Published:** 2021-02-26

**Authors:** Aleksandr Gorst, Kseniya Zavyalova, Aleksandr Mironchev

**Affiliations:** Radiophysics Faculty, Tomsk State University, 634050 Tomsk, Russia; gorst93@gmail.com (A.G.); mironchev42@mail.ru (A.M.)

**Keywords:** sensor, combined slot antenna, diabetes, dielectric permeability, electromagnetic fields, glucose concentration, near-field sensor, non-invasive measurements, microwave sounding

## Abstract

The article presents a model of a near-field sensor for non-invasive glucose monitoring. The sensor has a specific design and forms a rather extended near-field. Due to this, the high penetration of electromagnetic waves into highly absorbing media (biologic media) is achieved. It represents a combined slot antenna based on a flexible RO3003 substrate. Moreover, it is small and rather flat (25 mm in diameter, 0.76 mm thick). These circumstances are the distinguishing features of this sensor in comparison with microwave sensors of other designs. The article presents the results of numerical modeling and experimental verification of a near-field sensor. Furthermore, a phantom of human biological media (human hand) was created for experimentation. In the case of numerical modeling, the sensor is located close to the hand model. In a full-scale experiment, it is located close to the phantom of the human hand for the maximum interaction of the near-field with biological materials. As a result of a series of measurements for this sensor, the reflection coefficient is measured, and the dependences of the reflected signal on the frequency are plotted. According to the results of the experiments carried out, there is a clear difference in glucose concentrations. At the same time, the accuracy of determining the difference in glucose concentrations is high. The values of the amplitude of the reflected signal with a change in concentration differ by 0.5–0.8 dB. This sensor can be used for developing a non-invasive blood glucose measurement procedure.

## 1. Introduction

The number of people with diabetes is growing every year. Back in 2002, Jones M. and Harrison J. M. described in their article [1] that the World Health Organization (WHO) expects an increase in the number of people with diabetes to 300 million by 2025. According to a survey conducted by the International Diabetes Federation (IDF) already in 2013, three-hundred eighty-two million patients worldwide had diabetes. Seven years later, in 2020, the number of people living with diabetes was 463 million. Diabetes mellitus is a serious disease that can lead to many complications. There are several types of the disease. The main types are type 1 and type 2 diabetes. They are the most prevalent. In both of these cases, a person with diabetes needs to control blood sugar levels to avoid the complications of the disease. Self-testing devices require small blood sampling using different needles, which cause pain and discomfort to the user of the device. Today, a large number of methods and devices are being developed for determining glucose levels using non-invasive and continuous methods. This approach will allow people with diabetes to avoid the discomfort of measuring blood sugar and monitor it throughout the day. The most well-known non-invasive methods for measuring blood glucose are Raman spectroscopy, impedance spectroscopy, near-infrared spectroscopy, photoacoustic spectroscopy, and others.

Raman spectroscopy [2,3] is based on the measurement of scattered light. The disadvantages of this method are the instability of the intensity and wavelength of laser beams during probing, a long time for obtaining data, as well as errors associated with chemical substances in the tissues. Impedance spectroscopy is based on the measurement of resistance when the radiation frequency changes. To measure the glucose level, several sensors located in the area of the veins in human hands are needed [4]. Near-infrared spectroscopy is based on the transmission of near-infrared radiation through a vascular region of the body (finger, earlobe, etc.). In this case, the glucose concentration is calculated on the basis of the received spectral information [5,6]. All measurements in near-infrared spectroscopy are based on the transmission of light through or into the sample and the measurement of the intensity (transmitted or reflected) of the beam. Spectrometers for measurements in near-infrared spectroscopy have a suitable light source (such as a highly stable quartz tungsten lamp), a monochromator or interferometer, and a detector. Conventional monochromators are acousto-optic tunable filters, diffraction gratings, or prisms. Mid-infrared spectroscopy is based on the absorption of light by glucose molecules [7,8,9]. This method uses a beam of light to travel through a crystal in contact with the skin. Thus, the electromagnetic field generated by the reflected light reaches the dermis (the layer of skin that contains the most glucose). The disadvantage of this method is the dependence of the obtained data on the water content in the dermis. Therefore, the reliability of the results largely depends on the degree of hydration. There is a technology based on ultrasonic sensing: photoacoustic spectroscopy [10,11]. This method is based on the acoustic response of a liquid using laser light. This method is similar to mid-infrared spectroscopy. There are also other less known methods for determining the level of glucose in the blood [12,13].

With the existing variety of physical methods used for the non-contact determination of blood glucose concentration, the problem of creating non-invasive glucometers has not yet been solved. At the same time, conventional invasive glucometers have been actively improving over the past twenty years: chemical analysis has been replaced by electrochemistry; the devices are equipped with internal memory and other conveniences; their use has become easier, but still painful. It should be noted that invasive analysis is a direct method; in this case, a sample (blood drop) containing glucose is directly examined. While analyses of non-invasive glucometers are indirect methods, they are based on data obtained, as a rule, by spectral means. In some cases (IR spectroscopy), an attempt is made to quantitatively analyze blood glucose without removing a sample from the body; in others (for example, electrical and thermal characteristics), the study of factors associated with glucose levels is carried out in a very complex and ambiguous way. In any situation, the influence of surface tissue structures, the individual characteristics of the skin and the composition of the intercellular fluid, the functional complexity of blood components, and many difficult parameters of the external and internal environment are very great. The glucose percentage is a continuously changing parameter.

At the same time, in recent years, many laboratories and companies have been developing non-invasive glucometers, and some of the ready-made solutions have even received certification. In 2014, French researchers Chretiennot T., Dubuc D., and Grenier K.proposed a resonant microwave biosensor, which, according to the authors, achieved the accuracy of chemical glucometry methods. They published the paper [14]. However, this method cannot be rightfully considered non-invasive: it still requires the extraction of physiological fluids for analysis. A glucometer in the form of a patch (sugarBEAT) was created by the British company Nemaura Medical and certified in Europe. The sugarBEAT patch is only 1mm thick. It measures the glucose level in the tissue fluid of the upper layer of the skin (in sweat) and transmits the measurement results every 5 min via a digital interface. It is worth noting that sugarBEAT does not relieve the traditional finger puncture procedure, but it only needs to be done once to calibrate the meter before attaching it. The term of its operation can be up to two years. The American company M10, together with Seoul National University, created a prototype of a wearable device. It can not only measure the level of glucose in the blood, but also administer the required dose of insulin. The small patch contains sensors for glucose, temperature, humidity, and pH (sweat particles are used for measuring), as well as microneedles for injecting the drug and a heater (with its help, the needles are activated).

The SugarSenz Velcro glucometer of the American company Glucovation is attached to the stomach and constantly measures the glucose level with the possibility of digital data transmission. However, it also pierces the skin, not like conventional glucometers, but to the subcutaneous layer, in which the glucose level can also be measured. Google X lab worked on the creation of contact lenses, which, using a special sensor, could measure glucose levels not in blood, but in tears. However, glucose in the extracellular fluid is different from blood glucose. On average, the physicochemical reaction between glucose and the electrode surface takes three to 20 min. During this time, glucose passes from the blood into the intercellular fluid, and only then, the received signal undergoes algorithmic processing. Therefore, such devices are suitable for detecting trends in sugar levels, but are not suitable for the instant and accurate analysis required in real conditions. GlucoTrack DF-F is a non-invasive blood glucose meter of the Israeli company Integrity Applications (received a certificate from the European Commission). It uses three ways to measure blood sugar: ultrasonic, electromagnetic, and thermal. All of these measurements are taken using a miniature clip-on transducer that clings to the ear. With three measurements, the meter can give a more accurate reading. The disadvantages are the need for regular device calibration and the lack of accuracy.

Scientists at the Swiss Institute in Lausanne (EPFL) have created a miniature implant that can measure various parameters of a person’s blood, such as glucose or cholesterol levels. The sensor is installed under the skin, and above it, a small unit with a battery and a wireless transmitter is attached to the skin. C8 MediSensors (a non-invasive blood glucose meter of the American company C8 MediSensors (European certificate)) is based on the optical principle and does not require a blood sample. The meter sensor is attached to the skin in the abdomen and transmits measurements digitally. The measurement uses Raman spectroscopy technology.

GlucoWatch is a glucometric watch developed by Cygnus Inc (Petoskey, MI, USA). The sensing element is a sensor in contact with the skin. Using a weak electrical current, the sensors pull glucose out of the skin cells. The glucose entered into the sensor is measured and converted into blood glucose, and the result is displayed on the screen and, together with the date and time of the analysis, is recorded in the device’s memory. Measurements are taken every 10 min for 13 h. The tests revealed the following: (1) the device is inconvenient in operation (the manual is a book of 112 pages) and expensive to maintain; its use requires a complex individual calibration; it is not suitable for some types of skin; the analysis area on the hand must change every time; (2) the data obtained with its help are less accurate than measurements on a conventional glucometer; in some cases, the error can reach 25–30%; (3) the change in the level of glucose in the cell fluid occurs with a delay in relation to the change in blood glucose, and as a result, GlucoWatch lags behind the true state of affairs by ten or more minutes.

Omelon V-2 is a technology developed in Russia. The principle of its action is based on the fact that muscle and vascular tone are dependent on glucose levels. The device measures the pulse wave, vascular tone, and blood pressure several times, based on which it calculates the sugar level. The high percentage of coincidences of the calculated indicators with laboratory data allowed this blood glucose monitor to be launched into mass production. However, the device has dimensions of 155 × 100 × 45 cm, which does not allow carrying it in a pocket, and the correctness of the readings depends on the observance of the rules for measuring pressure: the correspondence of the cuff to the girth of the arm, the patient’s calmness, and the absence of movement during the operation of the device.

The Israeli firm Integrity Applications solved the problem of painless, fast, and accurate blood sugar measurement by combining ultrasonic, thermal, and electromagnetic technologies in the GlucoTrack DF-F glucometer model. The GlucoTrack Model DF-F is intended for use by adults (over 18 years of age) with type 2 diabetes. The GlucoTrack Model DF-F is an expensive monitoring device and cannot be used for diagnostics.

The Symphony tCGM System, developed by Echo Therapeutics (Franklin, MA, USA), measures sugar levels transdermally. However, for the correct installation of the sensor and its accurate operation, it is necessary to pre-treat the skin with a special device, the Prelude SkinPrep System; it performs a superficial peeling of the skin area on which the study will be carried out by improving the electrical conductivity of the skin. After preparation, a sensor is attached to the skin area, and after a while, the device displays data, including indicators of glycemia and the percentage of body fat. This information can also be transferred to a smartphone. The accuracy of the device reaches 95%, which is slightly lower than that of standard invasive glucometers. This technology is under development.

Japanese startup Quantum Operation unveiled at CES 2021 a smartwatch-style device that is supposedly capable of accurately measuring blood glucose without puncturing the skin. The principle of action is based on spectroscopy (Raman spectroscopy). Judging by the information from the sources, the watch does not constantly monitor the glucose level, but does it after activating the corresponding function, and one must wait for about 20 s for the result. This is less effective than real-time monitoring, which is provided, for example, by the well-known partially invasive FreeStyle Libre device.

The large number of technologies presented to date for non-invasive glucometers shows the extreme popularity of the research area (only a few of them are noted above). However, despite the variety of methods being developed, a device that meets all the necessary requirements such as complete non-invasiveness, the accuracy of readings within the permissible error (laboratory tests are taken as basic ones, and it is believed that the readings of the glucometer should not differ from them by more than 10–15%), ease of use, lack of consumables, price, etc., has not yet been created. Thus, the development of a non-invasive method for the determination of glucose remains an urgent task today. This article is devoted to the development of a sensor based on near-field microscopy. The near-field method provides deep penetration of electromagnetic waves even in a highly absorbing environment, which, in particular, is human biological tissue.

The work is organized as follows. At the beginning of the work, a model of the sample of the biological medium in the form of a human hand is calculated, and the dependences of the real part of the dielectric permittivity of various concentrations of glucose in physiological saline on the frequency are plotted based on the experiment. This is done to bring numerical simulations closer to real conditions. Next, a numerical simulation of the sensor is carried out, based on which a real model of the sensor is created. Furthermore, a flat-layered phantom of a human hand is created for the experiments. Then, the analysis and results of numerical and field experiments of the proposed sensor design are presented.

## 2. Materials and Methods

### 2.1. Hand Model

To develop a new sensor for non-invasive diagnostics of glucose levels, it is necessary to take into account several factors that can influence the measured value of the reflected electromagnetic signal. It should also be borne in mind that the concentration of glucose in different tissues of a human is distributed differently; the thickness of tissue layers of different people is different; and these are previously unknown values for non-invasive measurements. When developing non-invasive methods for measuring glucose by electromagnetic methods, first of all, it is necessary to consider the effect of skin layers.

This is important because skin, like blood, has a high dielectric permittivity. Therefore, the electromagnetic wave attenuates in it more strongly than in other layers. Each layer of the skin has its dielectric characteristics, which may differ from human to human due to differences in the morphology and thickness of skin layers, the concentration of tissue/blood components (such as glucose), cutaneous blood perfusion, etc.

The skin has a great influence on glucose measurement; it can vary in the range of 2–10 mm when looking at the whole human body. The skin has a multilayer structure and includes stratum corneum; its thickness ranges from 15 to 150 microns in various parts of the body; the epidermis, the upper outer layer of human skin, which includes about 15–35% interstitial fluid, without blood vessels; the dermis, the skin itself, is a connective tissue and contains arterioles, venules, capillaries, and about 40% interstitial fluid. Furthermore, the dermis includes subcutaneous fatty tissue and loose fibrous connective tissue. The distribution and thickness of the skin depend on heredity, sex hormones, and human living conditions. On average, the thicknesses vary in the following ranges: the epidermis thickness is 0.068–0.146 mm; the stratum corneum of the epidermis is 0.021–0.049 mm; dermis is 1.89–3.04 mm; the subcutaneous fat is 0.03–1.41 mm. The next layer is subcutaneous fat, which has a wide range of thicknesses. The thickness of the fat layer on the forearm is the smallest compared to other parts of the body. Before considering muscle fibers, it is required to consider the saphenous veins passing through the human forearm.

To measure glucose with a standard glucometer, blood is drawn from the subcutaneous capillary vessels. In a medical examination, blood is drawn from a vein. The fundamental difference between these two methods is measurement accuracy. It should be understood that in medicine, the level of glucose (glycated hemoglobin) is determined in practice by venous blood. The content of these substances in venous and arterial blood is somewhat different. For a more accurate calculation of the concentration of glucose, the venous blood was considered in our study. A vein has a multi-layered structure. Depending on the type of vessels, they have different thicknesses, densities, and permeabilities. Large vessels additionally contain small blood and lymphatic capillaries.

Due to different pressures during the entire period of a person’s life, the blood supply (blood volume in the measurement area) changes; along with this, the diameter of the vessels also changes. People with symptoms of tachycardia (increased heart rate) have smaller vessels because a rapid heartbeat decreases the efficiency of the heart since the ventricles do not have time to fill with blood. As a result, blood pressure decreases, and blood flow to organs decreases; hence, the area for the microwave signal response is smaller.

**Figure 1:**
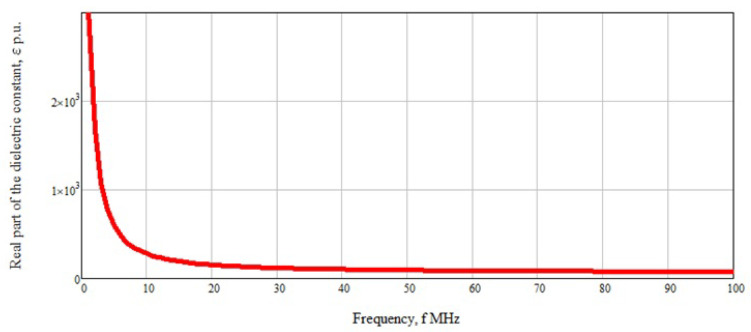
Dependence of the real part of the dielectric permittivity on frequency.

Another factor that can affect the dielectric properties of blood is blood hematocrit. Blood hematocrit refers to the percentage of red blood cells in the blood. Differences in the size, morphology, and distribution of red blood cells in human blood lead to changes in its dielectric properties, regardless of glucose concentration, and thereby affect the accuracy of glucose determination using measurement methods based on dielectric properties. Normally, this indicator is 40–48% for men and 36–42% for women. At low frequencies (about 3 MHz), the spread in the dielectric permittivity is quite high in the region of 30% with a change in hematocrit of 5% [15]. This scatter is associated with a rapid change in blood dielectric permittivity from frequency, which is clearly shown in Figure 1. With increasing frequency, this effect disappears due to the linear behavior of the dependence of the real part of the dielectric permittivity. In this case, the change will vary by 0.01–0.02%.

Based on the results of the literature review and the analysis of the studied information, the summary Table 1 was formed. It shows the thicknesses of all materials used for modeling.

**Figure 2:**
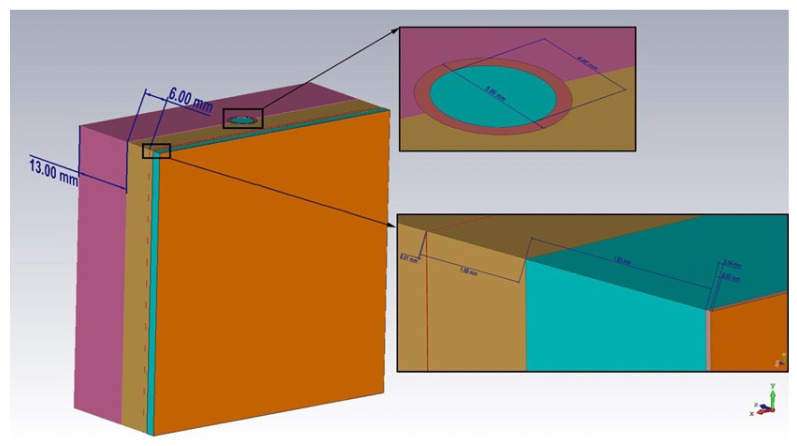
Model of the sample of the biological medium in the form of a human hand.

The values for the thicknesses of the muscles and bones of the human forearm are not given in the table, since when the near-field penetrates these tissues, a strong signal attenuation occurs, and the response from them will be at the noise level. Therefore, these layers in the calculation of the thicknesses of the materials used in the simulation can be neglected. Based on the given data, we created the model of a biological medium sample in the form of a human hand (Figure 2).

The model has a flat-layered structure. All previously described materials were modeled, and a 13 mm thick muscle layer was added. We used a flat structure because the used antenna substrate is flexible. In the future, when creating a real prototype of the sensor, this will make it possible to attach it directly to the area of human skin.

**Figure 3:**
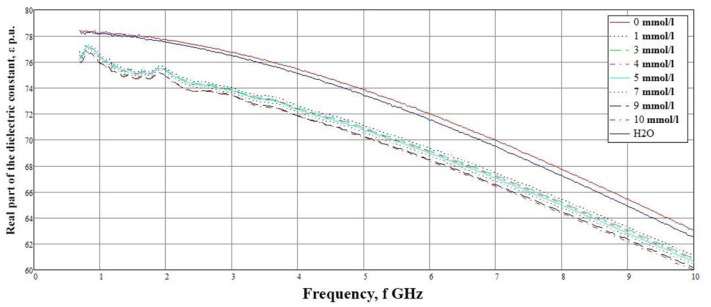
Dependence of the real part of the dielectric permittivity of solutions on frequency.

In the hand blood model, we used the data of the dielectric permittivity of saline with the following glucose concentrations: 0, 1, 3, 4, 5, 7, 9, and 10 mmol/L (Figure 3).

The data were analyzed using a PNA-L Network Analyzer (N5230C) and Dielectric Probe Kit-85070E Slim Form from Agilent Technologies. In subsequent simulations for blood with varying glucose concentrations, we used data obtained experimentally for physical solutions (saline) with different dextrose concentrations. Thus, it was possible to bring numerical modeling closer to real conditions.

### 2.2. Sensor Design

**Figure 4:**
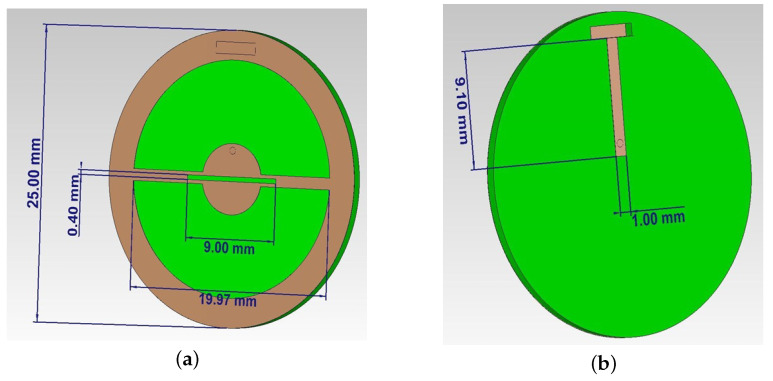
Near-field sensor model. Front (**a**) and back (**b**) views.

The development of the near-field sensor was based on a combined slot antenna. The brown color in Figure 4 indicates the ideal conductor and green the flexible dielectric RO3003 with relative permittivity ε = 3. The shape of the sensor resembles a coin in the center of which there is a metalized circle with a slot in it. Then, using two rectangular metalized sections, the circle is connected to a conductive frame (Figure 4a). The backside of the sensor is a dielectric layer with a microstrip line close to a central circle and a small cut in the dielectric up to the conductive frame for connecting to the supply element (Figure 4b). The probing near-field is formed on the side with a slot (Figure 4a). Due to the presence of several radiating elements (slit and frame), the formation of the reactive parts of a specific interference energy flow takes place. These features can increase the sensor’s sensitivity for near-field diagnostics of biological media and objects.

**Figure 5:**
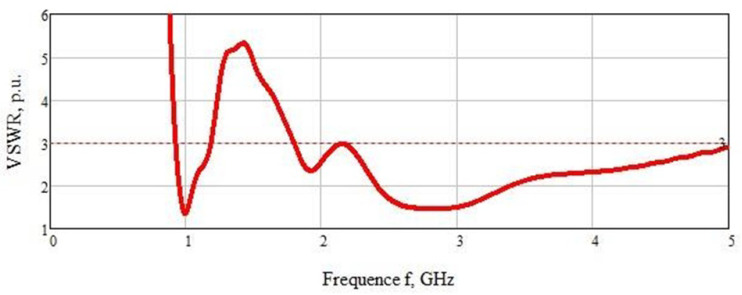
VSWRsensor coherence with the sample of the biological medium.

The VSWRof the sensor is also considered in terms of coherence with a sample of a biological medium in the form of a model of a human hand (Figure 5). The graph shows two sections of the curve with the highest matching of the sensor and the sample under study: the first at a frequency of 1 GHz (VSWR = 1.4) and the second in the range of 2.1–5 GHz (VSWR = 1.4). Note that almost the entire VSWR graph is below Level 3.

In the numerical simulation of the experiment to determine the concentration of glucose, the sensor was closely applied to a sample of a biological medium in the form of a human hand. The measurements were carried out in the frequency range of 0.5–5 GHz. We used this range to cover the entire frequency range in which the VSWR of the sensor is less than Level 3.

## 3. Experiments

### Creation of a Sensor and a Phantom of Human Biological Tissues

**Figure 6:**
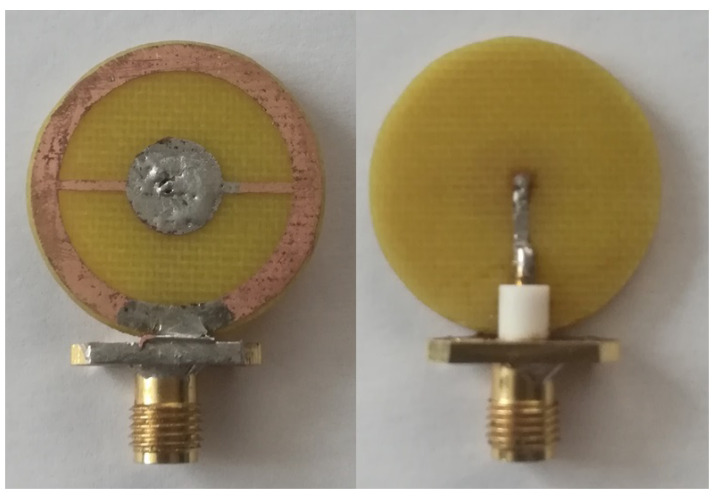
Photo of a near-field sensor.

The sensor production was based on a calculated model. We used the same materials as in the simulation: a Rogers3003 substrate was use;, the sensor was made with dimensions of 25 mm in diameter; and a 50 Ohm port was soldered to it. A photograph of the sensor is shown in Figure 6.

The production of the biological phantom was based on graphite, polyurethane, and acetone [16]. Such a structure is strong enough for creating thin materials such as stratum corneum, epidermis, and the capillary layer. The listed layers are the thinnest. The calculated data on the components for the phantom layers are presented in Table 2.

**Figure 7:**
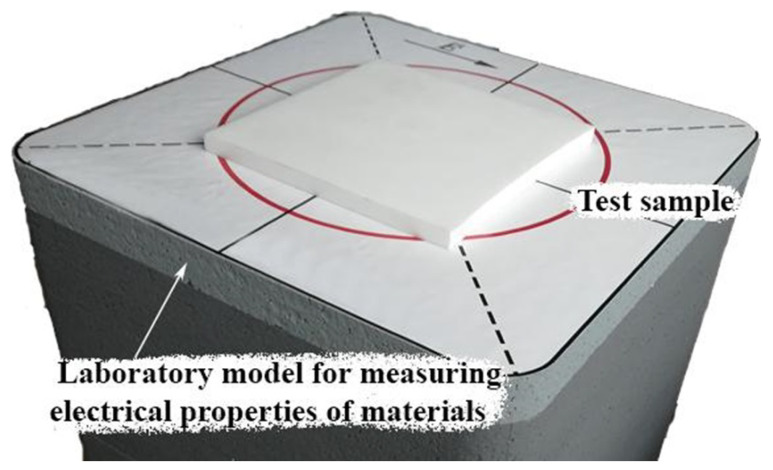
Installation for measuring the electrophysical parameters of the material [17].

The phantoms were made in the following way. Polyurethane HP40 and two-component polyurethane for forms were used in equal proportions. They were mixed according to the manufacturer’s instructions. Immediately thereafter, powder graphite and acetone were slowly added and mixed with the polyurethane base. The curing process took 12–16 h, but it was important to add the powders when the mixture was most flexible. The higher the mass percentage of the powder, the higher the dielectric properties of the sample were. However, the resulting mixture became lumpy when a large percentage of graphite was added. A small amount of acetone was added to ensure uniform mixing of each sample, as well as to create samples with a higher relative permittivity. The values of acetone affected the dielectric properties of the phantoms, so its amount was precisely adjusted to achieve the required values of the real part of the dielectric permittivity. The obtained flat materials were measured using a setup for measuring electrophysical parameters [17], as shown in Figure 7.

**Figure 8:**
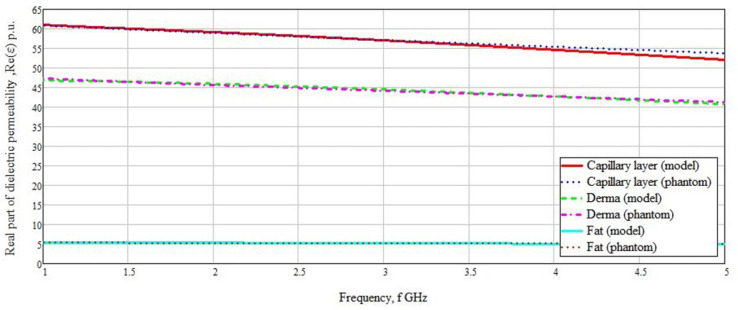
The real part of the dielectric permittivity of the model materials and the created phantoms.

**Figure 9:**
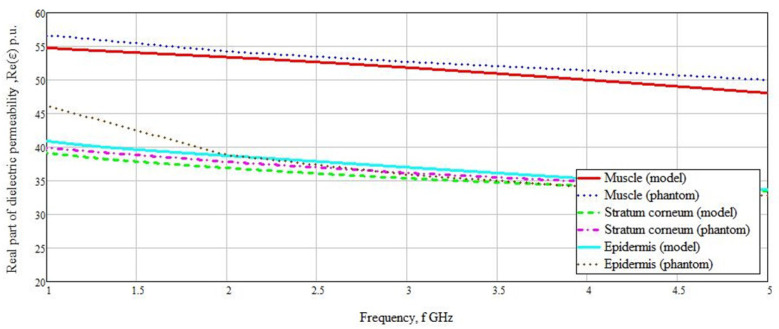
The real part of the dielectric permittivity of the model materials and the created phantoms.

Based on the measurement results, graphs of the real parts of the dielectric permittivity versus frequency were built for each of the materials (Figure 8 and Figure 9). As can be seen from the graphs, the values of the real part of the dielectric permittivity are similar to the materials presented in the simulation.

It can be observed that in Figure 8, the values for both the calculated and created materials match with high accuracy. A difference is seen for the capillary layer in the range from 4 to 5 GHz, but this range did not have much influence on the sensor measurements. The range we chose was 1–3 GHz. In this range, the difference in the values of the real part of the dielectric permittivity was minimal. The greatest difference in the values of the real part of the dielectric permittivity was observed in the muscles, stratum corneum, and epidermis.

The difference in the values of the real part of the dielectric permittivity averaged 1–2 rel.units. An abnormal case is presented by epidermal values. This deviation was in the frequency range we were measuring. Since the thickness of the epidermis does not exceed 0.1 mm, this value can be neglected because the reflected signal will undergo minimal changes. The graphs of stratum corneum and epidermis overlap because their values of the real part of the dielectric permittivity are similar in structure.

**Figure 10:**
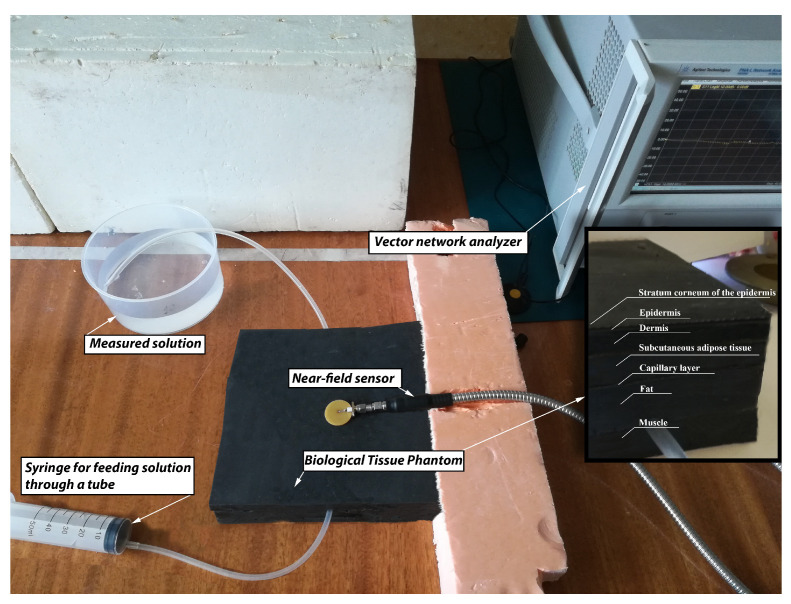
Manufactured graphite based phantom and the measuring setup for determining the level of glucose concentration in saline solution.

The used data made it possible to create a phantom of human biological tissues. The created area belongs to the forearm of the hand, because in this place lies a large in diameter vein. Each of the layers was made separately in different thickness molds. Subsequently, layers were cut into identical pieces of 150 × 150 mm in size (Figure 10). The size was selected based on the size of the antennas for measuring blood glucose concentration. The layers were layered sequentially. Due to the presence of acetone and graphite, each of the layers tightly adhered and glued to the other.

The measuring setup (Figure 10) was based on PNA-L Network Analyzer (N5230C) from Agilent Technologies. A silicone tube with an inner diameter of 5 mm was used as a venous vessel. A syringe was used to place saline of the required concentration of glucose inside the tube, and when the liquid was squeezed out, it passed through the tube, thereby simulating the flow of blood through the veins of a human. Such an approach allowed us to change the glucose concentration without physically affecting the antenna (transferring the antenna from one phantom to another), thereby making more accurate measurements.

## 4. Results and Discussion

### 4.1. Simulation Results

**Figure 11:**
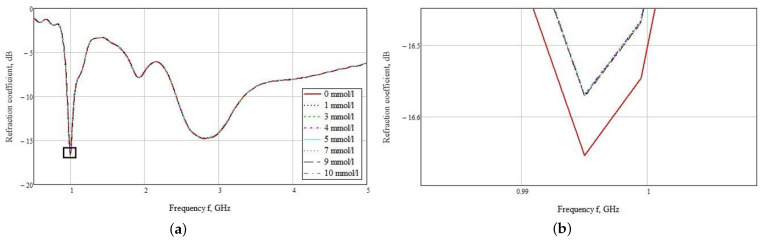
Frequency dependence of the reflected signal for a hand model with different glucose concentrations. Frequency range 0.5–5 GHz (**a**) and 0.5–1.5 GHz (**b**).

The used sensor was located close to the hand model for the maximum interaction of the near-field with biological materials. The dependences of the reflected signal on the frequency are plotted (Figure 11a) as a result of a series of measurements for this sensor model. You may notice that the graphs are poorly distinguishable. This is due to small changes in the dielectric permittivity. When zooming into a small area at 1 GHz, it can be seen that the most distinguishable reflected signal is observed for the hand model at 0 mmol/L blood glucose. The rest of the values differ only by a thousandth. Thus, the values for the concentration of 1 and 3 mmol/L are −16.672 and −16.671 dB, respectively (Figure 11b). In this graph, it is not possible to visually determine the various concentrations.

**Figure 12:**
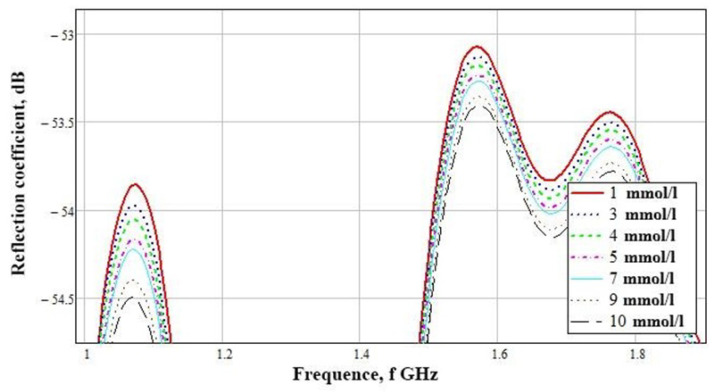
Parameter S11 for a model of a hand minus blood with a concentration of 0 mmol/L.

The subtraction of zero concentration was carried out, according to the result of which the graphs presented in Figure 12 were obtained. Differences are visible at the amplitude peaks of 1 GHz and the range of 1.5–1.8 GHz. Considering these graphs in more detail, we see a clear difference in concentrations at the frequencies presented above. To build a table with amplitude values, we used the maximum at 1.07 GHz.

Table 3 shows the numerical simulation data for a near-field sensor at a frequency of 1.07 GHz. This demonstrates its advantage in the accuracy of determining the concentration difference from the mean values. The difference of 1 mmol/L is 0.1 dB.

As a result of theoretical studies and numerical modeling, the design of a new combined sensor based on a resonant antenna and a near-field effect was proposed to determine the concentration of glucose in a biological medium in the form of a human hand.

### 4.2. Experimental Results

The simulation results showed that the developed sensor is able to distinguish between different levels of blood glucose concentration. The sensor was tightly attached to the test sample, and the simulation showed the best result at a frequency close to 1 GHz.

**Figure 13:**
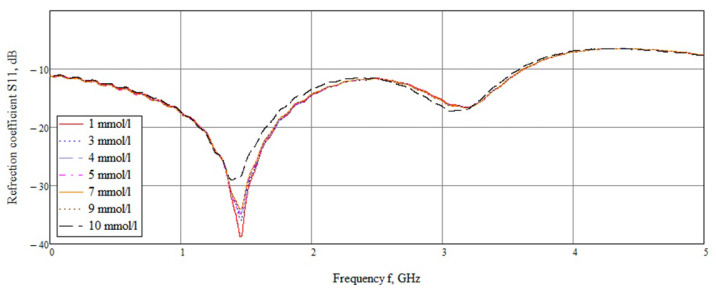
Dependence of the reflected signal on frequency for a near-field sensor in the frequency range 0–5 GHz.

An experimental study showed that the maximum difference in amplitudes is in the frequency range 1.45–1.55 GHz (Figure 13). This bias is due to the inaccuracy of the sensor manufacturing compared to simulation (1.07 GHz). Considering this range, it can be seen that the amplitudes at different concentrations vary.

**Figure 14:**
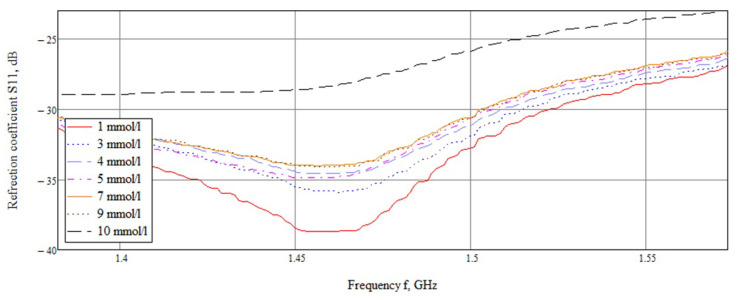
Dependence of the reflected signal on frequency for a near-field sensor in the frequency range 1.3–1.57 GHz.

A detailed examination (Figure 14) showed that the reflected signals are lined up in the correct sequence, but the difference between the values of 7 and 9 mmol/L is extremely small. This result is associated with the non-linear behavior of the dielectric permittivity in the frequency range of 1–2 GHz (Figure 3).

The rest of the concentrations are arranged in order, which indicates the correctness of this approach to the study of the concentration of glucose in the blood. Table 4 shows the average values of the amplitude of the reflected signal from the sensor for different glucose concentrations at a frequency of 1.53 GHz. Based on the results of the 10 conducted experiments, a confidence interval was constructed with errors for each individual glucose concentration value.

As can be seen from the table, the values of the amplitude of the reflected signal with a change in concentration differ by 0.15–0.4 dB; on average, the amplitude is 0.27 dB. The obtained sensitivity exceeded the simulation results (0.1 dB).

**Figure 15:**
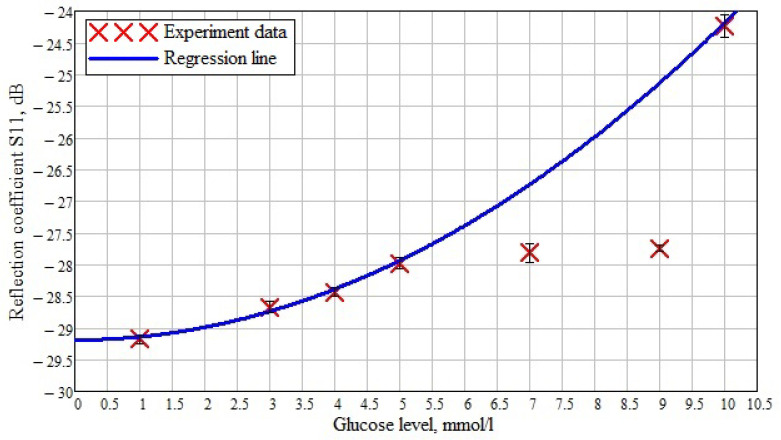
The dependence of the reflected signal on the concentration of glucose in saline at a frequency of 1.53 GHz.

Based on the obtained results, the dependence of the reflected signal (reflection coefficient S11) on various glucose concentrations in saline solution is plotted (Figure 15). The crosses mark the data obtained experimentally at a frequency of 1.53 GHz. A regression line is drawn along these experimental data, and an error interval was built for them taking into account the data presented in Table 4. It can be seen that at the minimum glucose concentration, the obtained values are aligned according to the regression curve. Furthermore, the concentration of 10 mmol/L matches the regression curve. In the case of concentrations of 7 and 9 mmol/L, the values differ from the expected ones, so the maximum difference is seen at a sugar level of 9 mmol/L. This deviation is associated with the non-linear behavior of the dielectric constant value at low frequencies (Figure 3).

Below is a comparison of the data obtained using the developed sensor with other developments (Table 5). To compare the results of this study with others, we selected the results where both the reflected signal and the transmitted signal were used as the measured parameters.

In [18], a microstrip glucose sensor measured the reflected signal and the input impedance. The authors managed to achieve a sensitivity of 0.18 dB/(mg/dL) at a frequency of 1.4–1.9 GHz. In [19], a method based on the change in the reflection coefficient between the dielectric waveguide resonator and the measured liquid was used. The proposed sensor allows detecting changes of 0.5 mg/mL at a sensor sensitivity of 0.003 dB/(mg/mL). Among the presented sensors from Table 5, the most sensitive is the sensor of [21]. It is based on the measurements of the transmitted signal with resonant frequencies in the range of 50–70 GHz. This method is based on the shift of the resonant frequency. The sensitivity of the sensor was 0.8–1 dB/(mg/mL) in an experimental study and 7.7 dB/(mg/mL) in simulation. Comparing the obtained data with the literature data, we can talk about the high sensitivity of the presented sensor in comparison with others. The proposed near-field sensor for non-invasive glucose monitoring surpassed all sensors in Table 5 at its sensitivity, which was 1.7–3.4 dB/(mg/mL).

## 5. Conclusions

In this work, a sensor based on a combined slot antenna is demonstrated. This sensor has an extended near-field with a high penetration depth of the electromagnetic field. A study of the biological structure of the human hand in the elbow bend is carried out, and subsequently, a simplified numerical model is created. We also measure the reflected signal from the hand model with a shallow vein. The sensor gives a high echo response with a small change in the glucose level in saline. The obtained data indicate the possibility of determining the concentration of glucose in the blood with great accuracy. The average scatter of the reflected signal data for a change of 1 mmol/L is 0.1–0.15 dB, which is high for such a small change in glucose. The phantom created on the basis of polyurethane, graphite, and acetone shows a high similarity of the dielectric permeability with the mathematical calculations of biological media. The experimental data obtained using the manufactured sensor show a larger spread of the reflected signal with a small change in glucose level.

Much work is planned to verify the data obtained. In addition, it is necessary to improve the installation. It should be portable and mobile. That is, we will need to at least replace the bulky Agilent vector network analyzer with a compact version of the device. It will be necessary to develop the hardware and software of the device, which would allow for the collection and processing of data in real time. It will be necessary to conduct a series of preliminary measurements on volunteers to collect statistics indicating the presence of a correlation between glucose levels and the response to probing signals. We plan to conduct research on volunteers using the commercially available semi-non-invasive FreeStyle Libre blood glucose meter and at the same time using the developed sensor. Further work will be aimed at eliminating inaccuracies in the calculation and conducting experiments on humans with a comparison of the data obtained with the data of venous blood (laboratory analysis in a clinic). This approach will allow comparing the data on the level of sugar in venous blood and interstitial fluid and more accurately adjusting the developed sensor.

## Figures and Tables

**Table 1:** Thickness of the materials used in modeling.

Name of Materials	Thickness, mm
Stratum corneum of the epidermis	0.02
Epidermis	0.04
Dermis	1.83
Subcutaneous adipose tissue	1
Hand vein	4
Hand vein wall	0.5
Fat	6

**Table 2:** Calculated data for creating biological tissues.

Name of Materials	Polyuret. HP40 %	Two-Comp. Polyuret.%	Graphite, %	Acetone, mL/100 g
Muscle	30	30	32	6.8
Fat	40	40	20	0.00
Capillaries	30	30	33.8	6.2
Dermis	30	30	32.3	7.7
Epidermis	32	32	30	6.0
Stratum corneum
of the epidermis	32	32	29.4	6.6

**Table 3:** The results of modeling the amplitude of the reflected signal by the proposed new sensor for different glucose concentrations.

Glucose Concentration, mmol/L	Frequency, GHz	Amplitude, dB
1	1.07	−53.85
3	1.07	−53.97
4	1.07	−54.05
5	1.07	−54.16
7	1.07	−54.22
9	1.07	−54.39
10	1.07	−54.49

**Table 4:** The results of measuring the amplitude of the reflected signal by the proposed new sensor for different glucose concentrations.

Glucose Concentration, mmol/L	Frequency, GHz	Amplitude, dB
1	1.53	−29.179±0.07
3	1.53	−28.676±0.09
4	1.53	−28.434±0.06
5	1.53	−27.979±0.11
7	1.53	−27.814±0.15
9	1.53	−27.743±0.05
10	1.53	−24.239±0.18

**Table 5:** Comparison of the developed sensor with other modern sensors for determining glucose levels.

Reference	Concentration, mg/mL	Frequency, GHz	Sensitivity Parameter dB per mg/mL	S
[18]	0.78–50	1.4–1.9	$S_{11}$	0.18
[19]	0–300	2–2.5	$S_{11}$	0.003
[20]	0–3	60–80	$S_{12}$	0.23
[21]	0.7–1.2	50–70	$S_{12}$	0.8–1
[22]	40–200	2.5–6	$S_{12}$	0.01
This work	0–1.81	1.45–1.55	$S_{11}$	1.7–3.4

## Data Availability

Data sharing not applicable.

## References

[1] Jones M., Harrison J.M. (2002). The future of diabetes technologies and therapeutics. Diabetes Technol. Ther..

[2] Forst T., Caduff A., Talary M., Weder M., Brändle M., Kann P., Flacke F., Friedrich C., Pfützner A. (2006). Impact of environmental temperature on skin thickness and microvascular blood flow in subjects with and without diabetes. Diabetes Technol. Ther..

[3] Hanlon E., Manoharan R., Koo T., Shafer K., Motz J., Fitzmaurice M., Kramer J., Itzkan I., Dasari R., Feld M. (2000). Prospects for in vivo Raman spectroscopy. Phys. Med. Biol..

[4] Caduff A., Hirt E., Feldman Y., Ali Z., Heinemann L. (2003). First human experiments with a novel non-invasive, non-optical continuous glucose monitoring system. Biosens. Bioelectron..

[5] Khalil O.S. (1999). Spectroscopic and clinical aspects of noninvasive glucose measurements. Clin. Chem..

[6] Heise H. (1996). Non-invasive monitoring of metabolites using near-infrared spectroscopy: State of the art. Horm. Metab. Res..

[7] Gebhart S., Faupel M., Fowler R., Kapsner C., Lincoln D., McGee V., Pasqua J., Steed L., Wangsness M., Xu F. (2003). Glucose sensing in transdermal body fluid collected under continuous vacuum pressure via micropores in the stratum corneum. Diabetes Technol. Ther..

[8] Lipson J., Bernhardt J., Block U., Freeman W.R., Hofmeister R., Hristakeva M., Lenosky T., McNamara R., Petrasek D., Veltkamp D. (2009). Requirements for calibration in noninvasive glucose monitoring by Raman spectroscopy. J. Diabetes Sci. Technol..

[9] Roychoudhury P., Harvey L.M., McNeil B. (2006). At-line monitoring of ammonium, glucose, methyl oleate and biomass in a complex antibiotic fermentation process using attenuated total reflectance-mid-infrared (ATR-MIR) spectroscopy. Anal. Chim. Acta.

[10] Waynant R., Chenault V. (1998). Overview of non-invasive fluid glucose measurement using optical techniques to maintain glucose control in diabetes mellitus. IEEE LEOS Newsl..

[11] Khalil O.S. (2004). Non-invasive glucose measurement technologies: An update from 1999 to the dawn of the new millennium. Diabetes Technol. Ther..

[12] Gabbay R.A., Sivarajah S. (2008). Optical coherence tomography-based continuous noninvasive glucose monitoring in patients with diabetes. Diabetes Technol. Ther..

[13] Guo X., Mandelis A., Zinman B. (2012). Noninvasive glucose detection in human skin using wavelength modulated differential laser photothermal radiometry. Biomed. Opt. Express.

[14] Chretiennot T., Dubuc D., Grenier K. Double stub resonant biosensor for glucose concentrations quantification of multiple aqueous solutions. Proceedings of the 2014 IEEE MTT-S International Microwave Symposium (IMS2014).

[15] Hayashi Y., Brun M.A., Machida K., Lee S., Murata A., Omori S., Uchiyama H., Inoue Y., Kudo T., Toyofuku T. (2017). Simultaneous assessment of blood coagulation and hematocrit levels in dielectric blood coagulometry. Biorheology.

[16] Oliveira B.L., O’Loughlin D., O’Halloran M., Porter E., Glavin M., Jones E. (2018). Microwave Breast Imaging: Experimental tumour phantoms for the evaluation of new breast cancer diagnosis systems. Biomed. Phys. Eng. Express.

[17] Gorst A., Zavyalova K., Shipilov S., Yakubov V., Mironchev A. (2020). Microwave Method for Measuring Electrical Properties of the Materials. Appl. Sci..

[18] Huang S.Y., Yoshida Y., Inda A.J.G., Xavier C.X., Mu W.C., Meng Y.S., Yu W. (2018). Microstrip line-based glucose sensor for noninvasive continuous monitoring using the main field for sensing and multivariable crosschecking. IEEE Sens. J..

[19] Kim S., Kim J., Babajanyan A., Lee K., Friedman B. (2009). Noncontact characterization of glucose by a waveguide microwave probe. Curr. Appl. Phys..

[20] Hu S., Nagae S., Hirose A. (2018). Millimeter-wave adaptive glucose concentration estimation with complex-valued neural networks. IEEE Trans. Biomed. Eng..

[21] Omer A.E., Gigoyan S., Shaker G., Safavi-Naeini S. (2020). WGM-based sensing of characterized glucose-aqueous solutions at mm-waves. IEEE Access.

[22] Harnsoongnoen S., Wanthong A. (2017). Coplanar waveguide transmission line loaded with electric-LC resonator for determination of glucose concentration sensing. IEEE Sens. J..

